# Prediction Models for Acute Kidney Injury in Stroke Patients: A Systematic Review

**DOI:** 10.1002/brb3.71188

**Published:** 2026-01-07

**Authors:** Baihui Zhong, Yifan Du, Xinyi Wang, Xue Dong

**Affiliations:** ^1^ Department of Nursing Changchun University of Chinese Medicine Changchun Jilin Province China

**Keywords:** acute kidney injury, prediction model, stroke, systematic review

## Abstract

Introduction

To systematically identify and synthesize the research on prediction models for acute kidney injury (AKI) in stroke patients. Methods: CNKI, Wanfang, VIP, CBM, PubMed, Cochrane Library, Embase, and Web of Science were searched from inception to April 26, 2025. The fundamental characteristics of the included studies were extracted, including model construction, predictors, model performance, and presentation methods. Results: A total of 35 prediction models were identified in this systematic review, with area under the curve (AUC) values ranging from 0.428 to 1.000. Seven studies performed external validation. Common predictors included hypertension, serum creatinine levels, age, diuretic use, mechanical ventilation, and the National Institutes of Health Stroke Scale score (NIHSS). Conclusions: The risk prediction model for AKI in stroke patients still needs to be developed. Despite demonstrating promising predictive capability, the models exhibited significant performance variability and an overall high risk of bias. Future research requires standardized development and validation of models to develop reliable prediction tools with minimal bias and enhanced applicability.

## Graphical abstract

Predictive models can assess the risk of acute kidney injury following stroke. However, there remains a need to develop tools with lower bias and higher applicability in the future.

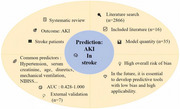

**Abbreviations**
AdaBoost: Adaptive Boosting
AKI: acute kidney injury
AKIN: Acute Kidney Injury Network
ASA: the American Society of Anesthesiologists grade
AUC: the area under the curve
DCA: Decision Curve Analysis
EPV: events per variable
GaussianNB: Gaussian Naive Bayes
GCS: Glasgow Coma Scale
Hosmer‐Lemeshow: test Hosmer‐Lemeshow goodness‐of‐fit test
ICU: Intensive Care Unit
KDIGO: Kidney Disease: Improving Global Outcomes
KNN: K‐Nearest Neighbors
LightGBM: Light Gradient Boosting Machine
MLP: Multi‐layer Perceptron
NIHSS: the National Institutes of Health Stroke Scale
SHAP: SHapley Additive exPlanations
SVM: Support Vector Machine
WBC: White Blood Cell
XGBoost: Extreme Gradient Boosting

## 1 Introduction

Stroke has emerged as the second leading cause of mortality worldwide. Research indicates an annual increase in both the global incidence and mortality rates of stroke, resulting in a significant economic burden on societies globally (GBD 2019 Stroke Collaborators 2021). AKI is characterized by a rapid decline in renal excretory function, with common etiologies including infection and hypovolemic shock (Kellum et al. 2021). AKI is a serious complication in stroke patients, and its development has been linked to post‐stroke physiological alterations in blood pressure, hormone levels, and treatment‐related factors (Arnold et al. 2018). About 8–21% of people with stroke develop AKI, potentially due to direct brain–kidney interactions and iatrogenic factors (Freeman et al. 2015; Khatri et al. 2014). AKI can significantly increase the mortality rate of stroke patients (Huang et al. 2020). Timely identification of AKI risk in this population is essential for improving clinical outcomes. Risk prediction models serve as valuable tools for the early detection of high‐risk groups, employing statistical methodologies that integrate various patient risk factors to estimate the likelihood of specific outcomes in defined populations (Collins et al. 2015). In recent years, numerous studies have introduced diverse risk prediction models for AKI. However, most of these models focus on specific populations, such as patients with sepsis or those undergoing cardiac surgery, while models explicitly designed for AKI after stroke remain largely exploratory (Feng et al. 2023). This study systematically reviews the existing literature on risk prediction models for AKI in stroke patients. It evaluates these models against current standards in the field (Shi et al. 2025), aiming to improve early identification and intervention of AKI post‐stroke.

## 2 Materials and Methods

This systematic review followed the PRISMA statement and registered with the International Prospective Register of Systematic Reviews (CRD420251081442).

### 2.1 Define the Research Question

Research Questions: What specific predictive models have been developed for AKI in stroke patients? What methods were adopted, and which predictors were selected to build these predictive models? How effectively do these predictive models perform in clinical practice?

### 2.2 Literature Search Strategy

CNKI, Wanfang, VIP, CBM, PubMed, Cochrane Library, Embase, and Web of Science were systematically searched from their inception to April 26, 2025, with references manually traced. MeSH terms, keywords, abstracts, or titles used for retrieval included “stroke” or “cerebral embolism” or “cerebral infarction” or “cerebrovascular accident” or “cerebral hemorrhage” or “ischemic stroke” or “hemorrhagic stroke,” “acute kidney injury” or “acute kidney injury” or “acute renal failure” or “acute renal insufficiency,” and “prediction” or “model” or “risk assessment” or “early warning” or “score” or “tool.” The detailed search strategy is presented in Table S1.

### 2.3 Criteria for Literature Inclusion and Exclusion

Inclusion criteria: Studies involving adult stroke patients (aged ≥18 years); that focused on the development or validation of risk prediction models for AKI in stroke patients; Study types included cohort studies, case‐control studies, and cross‐sectional studies; the outcome measure was the occurrence of AKI in stroke patients.

Exclusion criteria: Studies not written in English or Chinese; studies without full text available; studies focused exclusively on the analysis of risk factors without developing a predictive model for AKI; articles lacking valid data; reviews, dissertations, conference proceedings, and duplicate publications.

### 2.4 Literature Screening

**FIGURE 1:**
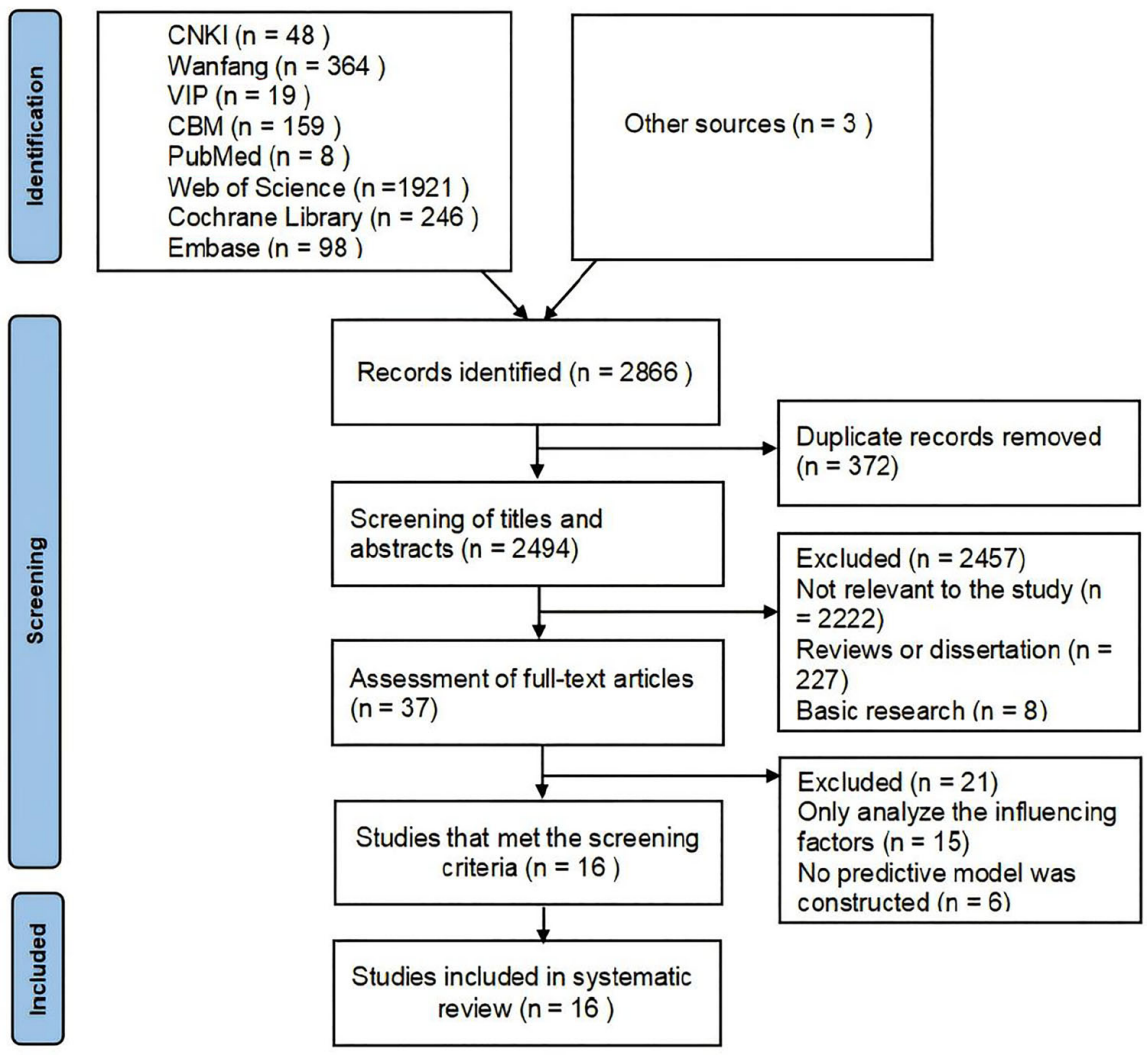
Retrieval and screening process flowchart.

The retrieved literature was imported into NoteExpress. Two researchers independently conducted the literature screening and information extraction, with any disputes resolved by a third researcher. The literature screening process is illustrated in Figure 1.

### 2.5 Evaluation of Methodological Quality

Two trained researchers used a predictive model bias risk assessment tool (PROBAST) to evaluate the bias risk and applicability of the included model (Chen et al. 2020). In the event of any disputes during the model evaluation process, a third researcher would be involved in the decision‐making.

## 3 Results

### 3.1 Literature Screening Results and Basic Characteristics of Included Literature

A total of 2,863 articles were retrieved during the initial examination. Subsequently, three studies were included from the retrospective literature, and ultimately 16 studies were included. The included articles, published from 2014 to 2024, originated from India (*n =* 1), South Korea (*n =* 1), and China (*n =* 14). The types of studies included consisted of cohort studies (*n =* 14) and cross‐sectional studies (*n =* 2). The fundamental characteristics of the included literature are summarized in Table 1.

**TABLE 1:** Basic characteristics of the included literature.

Author (year)	Country	Study type	Participants	AKI Definition	Data Source	Sample Size (cases)	Number of events	EPV	Model development method
Arora et al. 2024	India	cohort study	Stroke patients	KDIGO AKIN	A single tertiary care center	204	49 40	7, 4	Logistic Regression
Kim et al. 2014	Korea	cohort study	Hemorrhagic Stroke	AKIN	Samsung Medical Center of Korea	153	16	4	Logistic Regression
Zhang et al. 2022	China	cohort study	Patients with acute cerebrovascular disease	KDIGO	Intensive care units of a medical center and a hospital	3434	1467	146.7	XGBoost、AdaBoost, Random Forest, Logistic Regression, MLP
Liu et al. 2022	China	cohort study	Patients with ischemic stroke	KDIGO	US hospitals and medical center in Boston	4369	567	141.7	Logistic Regression
Lu et al. 2024	China	cohort study	Patients with cerebral infarction	KDIGO	The Intensive Care Unit and Emergency Medicine of a hospital	3920	1354	96.7	XGBoost, Logistic Regression, LightGBM, Random Forest, AdaBoost, GaussianNB, MLP, SVM, KNN
Ma et al. 2024	China	cohort study	Patients with ischemic stroke	KDIGO	Critical care database called MIMIC‐IV	2089	1452	121	Logistic Regression
Zhu et al. 2022	China	cohort study	Patients with acute ischemic stroke	KDIGO	Critical care database named MIMIC‐III	1132	171	19	Logistic Regression
Tian et al. 2023	China	cohort study	Patients with cerebral hemorrhage	KDIGO	Four independent medical centers of China	9649	4130	458.8	Logistic Regression
She et al. 2023	China	cohort study	Patients with cerebral hemorrhage	KDIGO	Critical care database named MIMIC‐III	1213	356	71.2	XGBoost, Logistic Regression, LightGBM, Random Forest, AdaBoost, SVM
Liu et al. 2023	China	cohort study	Patients with acute cerebral infarction	Expert Consensus on the Diagnosis and Classification of AKI	One hospital	100	61	20.3	Logistic Regression
Xue et al. 2024	China	cohort study	Patients with acute ischemic stroke	KDIGO	Two hospitals	805	110	13.7	Logistic Regression
Zhang et al. 2023	China	cross‐sectional study	Patients with acute ischemic stroke	KDIGO	One hospital	1633	238	23.8	Logistic Regression
Xiao et al. 2024	China	cohort study	Patients with cerebral hemorrhage	KDIGO	One hospital	207	35	11.6	Logistic Regression
Rao et al. 2022	China	cross‐sectional study	Patients with stroke	KDIGO	One hospital	1070	140	17.5	Logistic Regression
An et al. 2023	China	cohort study	Patients with acute ischemic stroke	KDIGO	One hospital	2177	146 230	24.3, 20.9	Logistic Regression
He et al. 2024	China	cohort study	Patients with cerebral hemorrhage	KDIGO	Critical care database named MIMIC‐III	997	620	62	LASSO Regression

Abbreviations: AdaBoost, Adaptive Boosting; AKIN, Acute Kidney Injury Network; EPV, events per variable; GaussianNB, Gaussian Naive Bayes; KDIGO, Kidney Disease: Improving Global Outcomes; KNN, K‐Nearest Neighbor; LightGBM, Light Gradient Boosting Machine; MLP, Multi‐layer Perceptron; SVM, Support Vector Machine; XGBoost, Extreme Gradient Boosting.

### 3.2 Model Quality Evaluation

**FIGURE 2:**
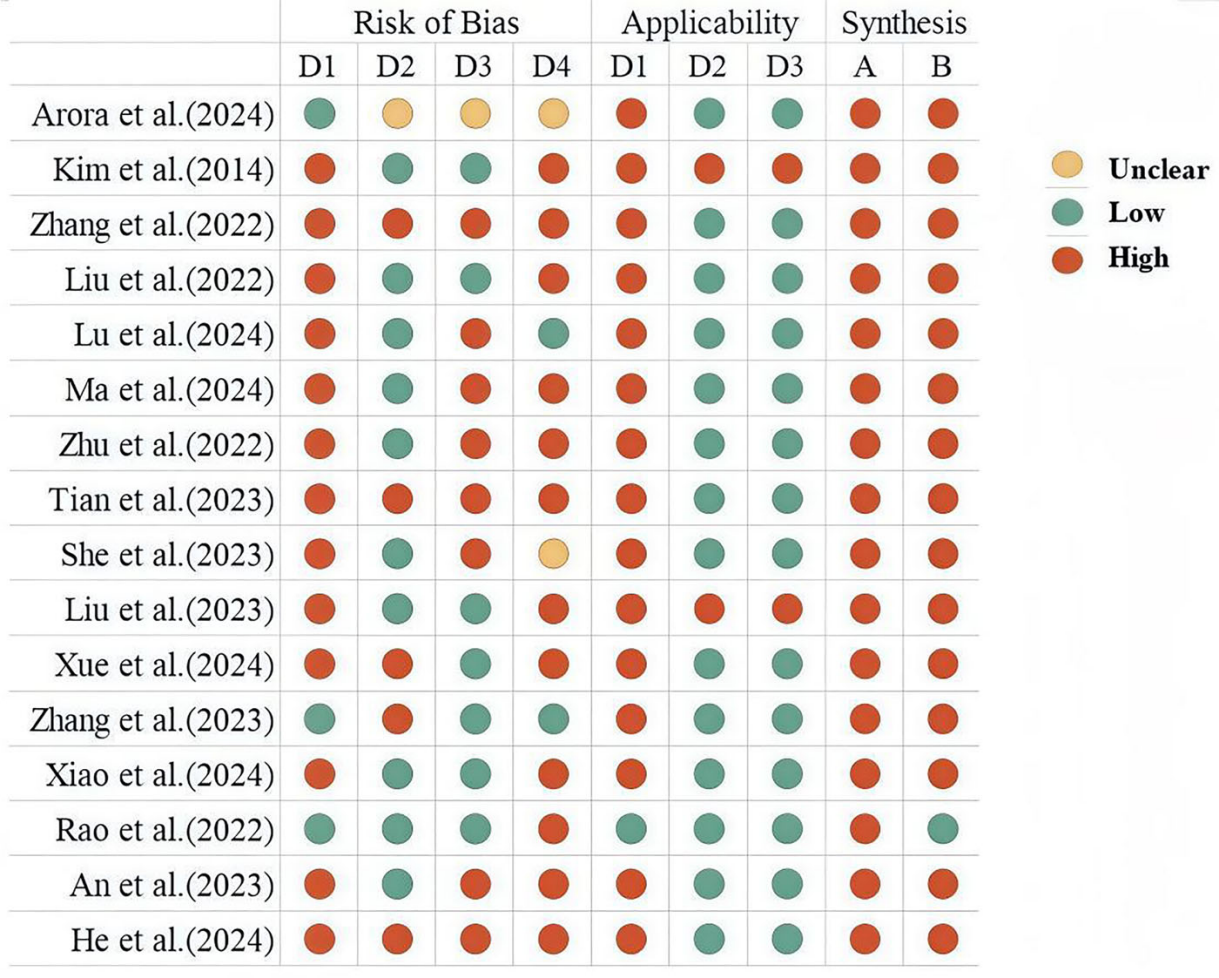
Risk of bias assessment results for included predictive models. *Note*: D1: Study Population; D2: Predictors; D3: Outcome; D4: Statistical Analysis; A: Risk of Bias; B: Applicability.

The risk of bias in the included prediction models was assessed using the PROBAST tool. All studies were evaluated as having a high risk of bias, particularly in the domains of participant selection and statistical analysis, as summarized in Figure 2. The item‐level rationales for the risk of bias assessment are provided in Table S2.

### 3.3 Basic Contents of the Prediction Model

#### 3.3.1 Model Construction

A total of 16 studies were included in this systematic review, and 35 predictive models were identified. The sample sizes ranged from 100 to 9,649 cases. The studies included are a cohort study (*n =* 14) and a cross‐sectional study (*n =* 2). The prediction models were constructed using multiple methods, including logistic regression (*n =* 15), random forest (*n =* 3), XGBoost (*n =* 3), AdaBoost (*n =* 3), SVM (*n =* 2), LightGBM (*n =* 2), MLP (*n =* 2), KNN (*n =* 1), GaussianNB (*n =* 1), and LASSO regression (*n =* 1).

#### 3.3.2 Model Predictors and Presentation Form

The number of predictors included in the 35 AKI prediction models for stroke patients varied significantly, ranging from three to 14. The commonly identified predictors were hypertension (*n =* 6), serum creatinine levels (*n =* 6), age (*n =* 6), diuretics use (*n =* 5), mechanical ventilation (*n =* 5), and the NIHSS score (*n =* 4). Thirteen studies reported the presentation forms of the models, mainly including nomograms, SHapley Additive exPlanations (SHAP), risk scoring formulas, calculators, and regression equations.

#### 3.3.3 Model Validation and Performance

The analysis included data from 16 studies. In this systematic review, 11 studies performed internal validation (Zhang et al. 2022; Lu et al. 2024; Ma et al. 2024; Zhu et al. 2022; Tian et al. 2023; She et al. 2023; Liu et al. 2023; Xue et al. 2024; Zhang et al. 2023; Xiao et al. 2024; He et al. 2024), while four performed both internal and external validation (Zhang et al. 2022; Lu et al. 2024; Tian et al. 2023; Xue et al. 2024), and three (Liu et al. 2023; Rao et al. 2022; An et al. 2023) focused exclusively on external validation. Additionally, two studies (Arora S et al. 2024; Kim MY et al. 2014) did not include any form of validation. The AUC values reported for the 27 models ranged from 0.428 to 1.000, with 24 models exceeding 0.75. Twelve studies employed specific reporting methods to evaluate model calibration. These methodologies included calibration curves (Zhang et al. 2022; Lu et al. 2024; Ma et al. 2024; Zhu et al. 2022; Tian et al. 2023; Xue et al. 2024; Zhang et al. 2023; He et al. 2024), the Hosmer‐Lemeshow goodness‐of‐fit test (Zhu et al. 2022; Tian et al. 2023; Liu et al. 2023; Xiao et al. 2024; Rao et al. 2022; An et al. 2023; He et al. 2024), and the Brier score (Ma et al. 2024; Xue et al. 2024). Detailed findings are presented in Table 2. For more detailed information about the model, please refer to Table S3.

**TABLE 2:** Model predictors and performance.

Author (year)	Predictors	Missing data handling	Model presentation	Model validation	Model performance	Calibration
Arora et al. 2024	Ischemic stroke: age, sex, mechanical ventilation, tracheotomy, history of hypertension, admission‐NIHSS, glomerular filtration rate Hemorrhagic stroke: age, sex, mechanical ventilation, tracheotomy, diabetes mellitus, history of hypertension, smoking, alcohol use, admission‐NIHSS, glomerular filtration rate	—	—	—	—	—
Kim et al. 2014	Age, hypertension, mannitol infusion rate, glomerular filtration rate	—	Rating scale	—	A:0.917(0.851‐0.983) B:—	—
Zhang et al. 2022	Serum creatinine levels, hemoglobin, WBC, bicarbonate, blood urea nitrogen, sodium, albumin, platelet count, age, length of hospital stay	Complete case analysis	—	Internal and external validation	A:— B:0.880/‐ 0.780/‐ 0.870/‐ 0.850/‐ 0.780/‐ 0.780/‐ 0.790/‐ 0.780/‐ 0.780/‐ 0.670/‐	Calibration curve
Liu et al. 2022	WBC to lymphocyte ratio, WBC to basophil ratio, WBC to hemoglobin ratio, neutrophil to lymphocyte ratio	—	Risk scoring formula	External validation	A:0.779 B:—	—
Lu et al. 2024	Drinking habits, respiratory rate, mechanical ventilation, serum creatinine levels, pulmonary infection, hemiplegia, diabetes mellitus, hypertension, NIHSS score, total cholesterol, low‐density lipoprotein, urea nitrogen, blood potassium, glomerular filtration rate	Multiple imputation	SHAP	Internal and external validation	A:1.000 0.962(0.954‐0.970) 1.000 1.000 0.971(0.964‐0.977) 0.933(0.922‐0.944) 0.903 (0.889‐0.917) 0.906 (0.892‐0.919) 0.938 (0.928‐0.948) B:0.955(0.937‐0.972) 0.955 (0.937‐0.973) 0.949 (0.930‐0.969) 0.941 (0.919‐0.963) 0.953 (0.935‐0.972) 0.933 (0.910‐0.955) 0.895 (0.866‐0.924) 0.911 (0.885‐0.937) 0.859 (0.825‐0.894)	Calibration curve
Ma et al. 2024	Weight, prior congestive heart failure, GCS, urine output, heart rate, blood glucose level, WBC, blood calcium concentration, vasoactive drug injection, furosemide administration, invasive mechanical ventilation, supplemental oxygen	Multiple imputation	Nomogram model	Internal validation	A:— B:—	Calibration curve and Brier Score
Zhu et al. 2022	Blood urea nitrogen, creatinine, red blood cell distribution width, heart rate, Oxford Acute Severity of Illness Score, the history of congestive heart failure, the use of vancomycin, contrast agent, mannitol	—	Nomogram model	Internal validation	A:0.8529(0.8036‐0.8954) B:0.8598(0.8017‐0.8806)	Hosmer‐Lemeshow test and calibration curve
Tian et al. 2023	Gender, systolic blood pressure, diabetes mellitus, GCS, mannitol infusion, serum creatinine levels, albumin, uric acid, and neutrophil/lymphocyte cell ratio	Complete case analysis	Calculators	Internal and external validation	A:0.815 (0.796‐0.833) B:0.816(0.788‐0.843) 0.776 (0.739‐0.814) 0.780 (0.745‐0.815) 0.821 (0.763‐0.878)	Hosmer‐Lemeshow test and calibration curve
She et al. 2023	Platelet count, serum creatinine levels, vancomycin level, hemoglobin level, hematocrit level	—	SHAP	Internal validation	A:0.846(0.816‐0.875) 0.698 (0.659‐0.737) 0.535 (0.500‐0.570) 1.000 0.810 (0.778‐0.841) 0.428 (0.383 ‐0.472) B:—	—
Liu et al. 2023	Age ≥60 years, comorbid hypertension, ultrasensitive C‐reactive protein ≥15.8 mg/L	—	Nomogram model	Internal validation	A:0.885 (0.800‐0.969) B:—	Hosmer‐Lemeshow test
Xue et al. 2024	Combined acute respiratory failure, elevated blood urea nitrogen, D‐dimer, monocyte count levels, antibiotics, diuretic use, mechanical ventilation, mannitol use	—	Nomogram model	Internal validation	A:0.877(0.844‐0.910) B:0.875(0.844‐0.911) 0.798(0.679‐0.917)	Calibration curve and Brier Score
Zhang et al. 2023	Elevated neutrophils, prolonged prothrombin time, elevated lactate dehydrogenase, decreased glomerular filtration rate, history of blood transfusion, comorbid chronic kidney disease, use of antibiotics, use of disulfiram, diuretic use, use of beta‐blockers	Multiple imputation	Nomogram model	Internal validation	A:0.797(0.769‐0.866) B:0.762(0.761‐0.762)	Calibration curve
Xiao et al. 2024	Preoperative GCS, ASA grade >3, heart rate at ICU admission	—	Nomogram model	Internal validation	A:0.795(0.727‐0.863) B:—	Hosmer‐Lemeshow test
Rao et al. 2022	Sex, history of hypertension, NIHSS score, history of collateral diuretic use, history of mechanical thrombolysis, serum β2‐microglobulin, urea nitrogen, serum cystatin C	Mean/Median Imputation	Regression equation	External validation	A:0.916(0.891‐0.940) B:0.906(0.853‐0.960)	Hosmer‐Lemeshow test
An et al. 2023	Younger group: Anemia, systolic blood pressure, homocysteine, alcohol consumption, blood urea nitrogen, NIHSS score Middle‐aged and older group: Hypertension, atrial fibrillation, previous history of stroke, cigarette smoking, infections, triglycerides, NIHSS score, use of antihypertensives, diuretic use, serum creatinine levels, blood urea nitrogen	—	—	External validation	A:0.938(0.912‐0.963) 0.838(0.808‐0.868) B:—	Hosmer‐Lemeshow test
He et al. 2024	Age, weight, heart rate, blood creatinine, invasive ventilation, vascular catheterization, heart failure, albumin, vancomycin medication use, GCS	—	Nomogram model	Internal validation	A:0.78 B:0.80	Hosmer‐Lemeshow test and calibration curve

Abbreviations: “–”, indicates not reported; A, development cohort; ASA, the American Society of Anesthesiologists grade; B, validation cohort; GCS, Glasgow Coma Scale; Hosmer–Lemeshow test, Hosmer–Lemeshow goodness‐of‐fit test; ICU, Intensive Care Unit; NIHSS, the National Institutes of Health Stroke Scale; SHAP, SHapley Additive exPlanations; WBC, White Blood Cell.

## 4 Discuss

### 4.1 The Predictors of the Risk Prediction Model for AKI in Stroke Patients Are Heterogeneous

Stroke is linked to an increased risk of AKI, a complication that is preventable. Assessing the potential risk of AKI in stroke patients is essential for early identification, which can significantly reduce the incidence of AKI and improve patient outcomes. The main predictors of AKI in stroke patients include hypertension, serum creatinine levels, age, diuretic use, mechanical ventilation, and the NIHSS score. The 12 most frequently identified predictors across the included studies are listed in Table S4. The included studies show significant heterogeneity in selecting predictors. This reflects differences in stroke types, patient characteristics, and key factors such as age. For example, the predictive models for AKI after cerebral hemorrhage incorporated preoperative GCS scores and the ASA grade, emphasizing specific clinical characteristics. In addition, inflammation and immune responses are linked to the pathological progression of ischemic stroke in the ICU setting. Therefore, the predictive model for AKI in ICU patients with ischemic stroke incorporated systemic inflammatory biomarkers (Zaid et al. 2019; Jin et al. 2013). Due to the differences in stroke types and treatment backgrounds, there is significant heterogeneity in the model predictors. Future research should assess the AKI risk of patients with different types of stroke, deeply explore predictive factors, and improve model performance for clinical application.

### 4.2 The Risk Prediction Models for AKI in Stroke Patients Still Need to Be Developed

All studies were evaluated as having a high risk of bias, particularly in the domains of participant selection and statistical analysis. In the participant domain, 13 studies were retrospective studies. Single‐center retrospective data may have problems such as incomplete data recording and selective bias, which can lead to deviations between the predicted results and the actual situation. Different demographic and clinical characteristics may lead to limitations in model performance and reduce its universality in other medical settings (Tang et al. 2020). It is necessary to conduct nested case‐control studies, prospective cohort studies, or other types of case‐control studies to reduce the risk of model bias caused by data sources.

In the statistical analysis domain, the overall risk of bias was evaluated as high. It may be attributed to the failure to report or properly handle missing data, the lack of comprehensive validation, and inadequate evaluation of model discrimination and calibration. The methods for handling missing values were not reported in ten studies. Data deficiency in medical records is common in clinical practice. If the predictive model does not include or directly excludes such cases, it may lead to inaccurate prediction results and increase the risk of misdiagnosis when applied by medical professionals. Furthermore, the studies included in this review have significant deficiencies in model validation: 11 studies performed internal validation, four performed both internal and external validation, three focused exclusively on external validation, and two studies did not perform any form of validation procedures. The lack of comprehensive verification may lead to inaccurate performance evaluation of the model, thereby limiting its true predictive ability. Different populations and geographical regions may lead to differences in the performance of prediction models (Zhang et al. 2024). In the community environment, the patients' conditions are relatively mild. Applying the prediction model developed based on the hospital environment to them may introduce bias. Therefore, future research should focus on conducting comprehensive validation of the prediction model to determine its effectiveness in different regions and medical environments.

Logistic regression analysis was used to develop prediction models in most of the included studies. This method is highly effective in dealing with discrete variables and has strong interpretability. However, logistic regression has limited ability in fitting nonlinear relationships and performs worse than complex models when dealing with large datasets. Compared with logistic regression, machine learning exhibits superior capabilities in variable selection and handling collinearity. This capacity to capture complex predictor‐outcome relationships enhances its utility in addressing intricate clinical challenges (Tran et al. 2024; Zhou et al. 2023). The comparative performance of traditional statistical methods and machine learning remains under debate. Future research should further explore the two methodologies and improve model construction techniques.

Calibration was reported in 12 studies, with eight utilizing calibration curves, seven applying the Hosmer‐Lemeshow test, and two employing the Brier score for evaluation. Improper model calibration may mislead clinical decisions and cause potential harm. The calibration of predictive models still needs further improvement. A significant number of the included studies did not report tools with clinical application value for the predictive models. Future research should prioritize developing readily implementable clinical tools to enhance their practical utility and accessibility. It has been shown in two studies that the AUC of the four models reaches 1.000. This apparently perfect performance is clinically unrealistic and indicates overfitting, which represents the main limitation of the research. The finding may be explained by methodological limitations, including insufficient sample size, data leakage, or inadequate validation. Future studies should strictly adhere to established reporting guidelines for predictive models, as well as the relevant criteria outlined in PROBAST. Increasing sample size, employing appropriate methodologies for handling missing data, and utilizing independent datasets for both internal and external validation are critical steps. Model evaluation should assess key performance aspects, including discrimination and calibration, to guard against overfitting or underfitting. The above steps play a fundamental role in improving the quality of research by enhancing the applicability and generalization ability of the model (Cao et al. 2020; Collins et al. 2015).

### 4.3 Implications for Nursing Practice and Research

Predictive models for AKI in stroke patients are crucial to optimize medical resource allocation and improve care quality (Lin et al. 2024). As primary caregivers, nurses play a critical role in identifying risk factors early and enhancing monitoring, which is pivotal for improving patient outcomes. However, most included studies did not provide an in‐depth discussion on the clinical implementation of these models, which may limit their practical utility and hinder iterative refinement. Future research should prioritize exploring the accessibility and applicability of prediction models in real‐world clinical settings. To facilitate this, nurses can integrate key and easily accessible predictors (for example, creatinine levels, NIHSS scores, and comorbidities) into the initial admission assessment form to facilitate initial risk screening and stratification. It is recommended that patients identified as high‐risk undergo consistent monitoring and dynamic evaluation. Nurses should accurately record urine output and fluid balance, avoid nephrotoxic medications, and closely monitor blood pressure. Any abnormalities should be promptly reported to the physician. Such systematic management can effectively enhance the quality of clinical decision‐making and reduce patient risks.

### 4.4 Checklists for Future Work and Clinical Adoption

(i) Minimum reporting checklist
  - Title and Abstract:

The manuscript should include a structured abstract, and the title must clearly specify whether the study focuses on the “Development” and/or “Validation” of a clinical prediction model.

  - Introduction:

The introduction should clearly state the study's objectives, specifying whether the aim is to develop a new prediction model and/or validate an existing model.

  - Methods:

Data Source: The study design, setting, participant inclusion criteria, and data collection period should be clearly described.

Outcome: The outcome measure should be precisely defined, and its assessment method should be described in detail.

Predictors: Clear definitions should be provided for all predictor variables, along with descriptions of their measurement or assessment methods.

Missing Data: Describe the methods employed to address missing data and report the quantity of missing values.

Model Development: The predictor selection process, statistical techniques for model development, and methods for evaluating model performance should be concisely summarized.

  - Results:

Participants: Include a flow diagram illustrating participant inclusion and exclusion, accompanied by a table summarizing baseline demographic and clinical characteristics.

Model Performance: Provide a comprehensive assessment of model discrimination (such as AUC) and calibration (such as calibration curve and calibration slope).

Final Model: Present the full model equation or regression coefficients to facilitate external validation and future application.

  - Discussion:

It is important to discuss the study's limitations and evaluate the potential clinical applicability and generalizability of the prediction model.

(ii) Clinical deployment checklist
  - External Validation: External validation across new populations, time periods, and geographical settings is essential to demonstrate the generalizability of the prediction model.
  - Model Performance: To comprehensively evaluate model performance, it is necessary to assess both discrimination (such as C‐statistic or AUC) and calibration, which reflects the alignment between predicted probabilities and observed outcomes.
  - Clinical Utility: Decision Curve Analysis (DCA) can be used to determine the clinical utility of the model. It demonstrates whether the model provides greater net benefit compared to “treat all” or “treat none” strategies across a range of clinically reasonable threshold probabilities.
  - Usability Tools: A detailed explanation should be provided regarding whether a clinically usable tool is available for the model, such as an online calculator or integration within electronic health record systems.
  - Handling of Missing Data: The methods used to address missing data should be clearly described, and the quantity of missing values should be reported.
  - Impact Assessment: High‐quality studies should be conducted to evaluate whether the application of the model leads to improved patient outcomes.
  - Prospective studies and model impact studies: Prediction models should be designed to integrate seamlessly into nursing routines, relying on easily collectible indicators. The primary evaluation focus should be whether their implementation genuinely leads to improved patient outcomes.

## 5 Limitations

This review has several potential limitations. Firstly, most of the included studies were conducted in China, which reflects the higher disease burden and clinical needs of AKI among stroke patients in this population. This may limit the generalizability of the findings to Western populations, necessitating adjustments to the predictive model for different regions. A key goal for future research is to develop and validate AKI prediction models that perform effectively across diverse stroke populations and healthcare settings. Secondly, as only studies published in English and Chinese were included in this review, there may be language bias, and findings published in other languages were not incorporated.

## 6 Conclusions

This study systematically evaluated the construction methods, predictors, and performance of AKI prediction models in stroke patients. The included studies have a high risk of bias. Future studies should incorporate a variety of model construction methods, perform comprehensive model validation, and select predictors based on both clinical relevance and empirical evidence. These steps will help provide clinical staff with more reliable and effective tools for identifying the risk of AKI in stroke patients.

## Author Contributions

**Baihui Zhong**: Writing – review and editing; writing – original draft; investigation; visualization. **Yifan Du**: Conceptualization; resources. **Xinyi Wang**: Formal analysis; methodology. **Xue Dong**: Methodology; supervision; funding acquisition.

## Funding

This research was funded by the Education Department of Jilin Province, grant number JJKH20250671KJ.

## Ethics Statement

We confirm that ethical approval was not required for this study because it is a systematic review of previously published data. No new data involving human participants were collected for this work.

## Conflicts of Interest

The authors declare no conflicts of interest.

## Supporting information



Supplementary Table: brb371188‐sup‐0001‐TableS1.docx

Supplementary Table: brb371188‐sup‐0002‐TableS2.docx

Supplementary Table: brb371188‐sup‐0003‐TableS3.docx

Supplementary Table: brb371188‐sup‐0004‐TableS4.docx

## Data Availability

Data sharing is not applicable to this article, as no new data were created or analyzed in this study. The data that support the findings are available in the manuscript.
