# Supplementary material for: Prediction Models for Acute Kidney Injury in Stroke Patients: A Systematic Review
Source: Brain Behav. 2026 Jan 7;16(1):e71188. doi: 10.1002/brb3.71188 (PMC12778413; doi:10.1002/brb3.71188)
Supplement: Supplementary file 1 — Supplementary Table: brb371188‐sup‐0001‐TableS1.docx [file BRB3-16-e71188-s003.docx]

**Supplementary Table 1** Literature search strategy

1. **Pubmed**

| Search number | Query | Results |
| --- | --- | --- |
| #1 | Stroke[Mesh Terms] | 190475 |
| #2 | Brain infarction[Title/Abstract] OR cerebral infarction[Title/Abstract] OR hemorrhagic stroke[Title/Abstract] OR ischemic stroke[Title/Abstract] OR cerebral hemorrhage[Title/Abstract] OR cerebrovascular accident[Title/Abstract] OR apoplexy[Title/Abstract] OR cerebral stroke[Title/Abstract] OR intracerebral hemorrhage[Title/Abstract] | 128872 |
| #3 | #1 OR #2 | 255342 |
| #4 | Acute Kidney Injury[Mesh Terms] | 60801 |
| #5 | Acute Renal Injury[Title/Abstract] OR Acute Renal Insufficiencies[Title/Abstract] OR Acute Kidney Insufficiencies[Title/Abstract] OR Acute Kidney Failures[Title/Abstract] OR Acute Renal Failures[Titl  e/Abstract] OR Acute Kidney Failure[Title/Abstract] | 2974 |
| #6 | #4 OR #5 | 62257 |
| #7 | Risk Assessment[Title/Abstract] OR machine learning[Title/Abstract] OR deep learning[Title/Abstract] OR predict model[Title/Abstract] OR prediction modeling[Title/Abstract] OR prognostic model[Title/Abstract] | 327717 |
| #8 | #3 AND #6 AND #7 | 8 |

**2.Cochrane**

| Search number | Query | Results |
| --- | --- | --- |
| #1 | MeSH descriptor: [Stroke] explode all trees | 17829 |
| #2 | (brain infarction):ti,ab,kw | 5438 |
| #3 | (cerebral infarction):ti,ab,kw | 6681 |
| #4 | (hemorrhagic stroke):ti,ab,kw | 2321 |
| #5 | (ischemic stroke):ti,ab,kw | 20854 |
| #6 | (cerebral hemorrhage):ti,ab,kw | 5784 |
| #7 | (cerebrovascular accident):ti,ab,kw | 18306 |
| #8 | (apoplexy):ti,ab,kw | 429 |
| #9 | (cerebral stroke):ti,ab,kw | 8720 |
| #10 | (intracerebral hemorrhage):ti,ab,kw | 3160 |
| #11 | #1 or #2 or #3 or #4 or #5 or #6 or #7 or #8 or #9 or #10 | 53689 |
| #12 | MeSH descriptor: [Acute Kidney Injury] explode all trees | 2436 |
| #13 | (Acute Renal Injury):ti,ab,kw | 4055 |
| #14 | (Acute Renal Insufficiencies):ti,ab,kw | 0 |
| #15 | (Acute Kidney Insufficiencies):ti,ab,kw | 1 |
| #16 | (Acute Kidney Failures):ti,ab,kw | 93 |
| #17 | (Acute Renal Failures):ti,ab,kw | 104 |
| #18 | (Acute Kidney Failure):ti,ab,kw | 7692 |
| #19 | #12 or #13 or #14 or #15 or #16 or #17 or #18 | 9898 |
| #20 | (Risk Assessment):ti,ab,kw | 84497 |
| #21 | (machine learning):ti,ab,kw | 3321 |
| #22 | (deep learning):ti,ab,kw | 1729 |
| #23 | (predict model):ti,ab,kw | 7189 |
| #24 | (prediction modeling):ti,ab,kw | 958 |
| #25 | (prognostic model):ti,ab,kw | 4728 |
| #26 | #20 or #21 or #22 or #23 or #24 or #25 | 97418 |
| #27 | #11 and #19 and #26 | 246 |

**3.Embase**

| Search number | Query | Results |
| --- | --- | --- |
| #1 | 'stroke'/exp OR stroke | 763997 |
| #2 | 'brain infarction':ab,ti OR 'cerebral infarction':ab,ti OR 'hemorrhagic stroke':ab,ti OR 'ischemic stroke':ab,ti OR 'cerebral hemorrhage':ab,ti OR 'cerebrovascular accident':ab,ti OR 'apoplexy':ab,ti OR 'cerebral stroke':ab,ti OR 'intracerebral hemorrhage':ab,ti | 193359 |
| #3 | #1 OR #2 | 797739 |
| #4 | acute AND kidney ANDinjury | 116815 |
| #5 | 'acute renal injury':ab,ti OR 'acute renal insufficiencies':ab,ti OR 'acute kidney insufficiencies':ab,ti OR 'acute kidney failures':ab,ti OR  'acute renal failures':ab,ti OR 'acute kidney failure':ab,ti | 4673 |
| #6 | #4 OR #5 | 118747 |
| #7 | 'risk assessment':ab,ti OR 'machine learning':ab,ti OR 'deep learning':ab,ti OR 'predict model':ab,ti OR 'prediction modeling':ab,ti OR  'prognostic model':ab,ti | 351283 |
| #8 | #3 AND#6 AND #7 | 98 |

**4.Web of science**

| Search number | Query | Results |
| --- | --- | --- |
| #1 | (((((((((TS=(Stroke)) OR TS=(brain infarction)) OR TS=(cerebral infarction)) OR TS=(hemorrhagic stroke)) OR TS=(ischemic stroke)) OR TS=(cerebral hemorrhage)) OR TS=(cerebrovascular accident)) OR TS=(apoplexy)) OR TS=(cerebral stroke)) OR TS=(intracerebral hemorrhage) | 1192980 |
| #2 | ((((((TS=(Acute Kidney Injury)) OR TS=(Acute Renal Injury)) OR TS=(Acute Renal Insufficiencies)) OR TS=(Acute Kidney Insufficiencies)) OR TS=(Acute Kidney Failures)) OR TS=(Acute Renal Failures)) OR TS=(Acute Kidney Failure) | 215078 |
| #3 | (((((TS=(Risk Assessment)) OR TS=(machine learning)) OR TS=(deep learning)) OR TS=(predict model)) OR TS=(prediction modeling)) OR TS=(prognostic model) | 5616034 |
| #4 | #1 AND #2 AND #3 | 1921 |

1. **Wanfang**

**Search Date:**April 26,2025

**Search Strategy:**

主题：（卒中 or 脑栓塞 or 中风 or 脑梗 or 脑血管意外 or 脑出血 or 缺血性卒中 or 出血性卒中）and 主题：（急性肾损伤 or 急性肾损害 or 急性肾功能衰竭 or 急性肾功能不全） and 主题：（预测 or 模型 or 风险评估 or 预警 or 评分 or 工具）

**Results:**A total of 364 records were initially retrieved.

**Translation:**

Subject：（Apoplexy or Brain infarction or Stroke or Cerebral infarction or Cerebrovascular accident or Intracerebral hemorrhage or Ischemic stroke or Hemorrhagic stroke）and Subject：（Acute Kidney Injury or Acute Renal Injury or Acute Renal Failures or Acute Renal Insufficiencies） and Subject：（Predict or Models or Risk Assessment or Early warning or Score or Tools）

**6.VIP**

**Search Date:**April 26,2025

**Search Strategy:**

M=（卒中 OR 脑栓塞OR 中风 OR 脑梗 OR 脑血管意外 OR 脑出血 OR 缺血性卒中 OR 出血性卒中） AND M=（急性肾损伤 OR 急性肾损害 OR 急性肾功能衰竭 OR 急性肾功能不全) AND M=（预测 OR 模型 OR 风险评估 OR 预警 OR 评分 OR 工具)

**Results:**A total of 19 records were initially retrieved.

**Translation:**

M=（Apoplexy OR Brain infarction OR Stroke OR Cerebral infarction OR Cerebrovascular accident OR Intracerebral hemorrhage OR Ischemic stroke OR Hemorrhagic stroke） AND M=（Acute Kidney Injury OR Acute Renal Injury OR Acute Renal Failures OR Acute Renal Insufficiencies) AND M=（Predict OR Models OR Risk Assessment OR Early warning OR Score OR Tools)

**7.CNKI**

**Search Date:**April 26,2025

**Search Strategy:**

（主题：卒中）OR（主题：脑栓塞）OR（主题：中风）OR（主题：脑梗）OR（主题：脑血管意外）OR（主题：脑出血）OR（主题：缺血性卒中）OR（主题：出血性卒中）AND（主题：急性肾损伤）OR（主题：急性肾损害）OR（主题：急性肾功能衰竭）OR（主题：急性肾功能不全）AND（主题：预测）OR（主题：模型）OR（主题：风险评估）OR（主题：预警）OR（主题：评分）OR（主题：工具）

**Results:**A total of 48 records were initially retrieved.

**Translation:**

（Subject：Apoplexy）OR（Subject：Brain infarction）OR（Subject：Stroke）OR（Subject：Cerebral infarction ）OR（Subject：Cerebrovascular accident）OR（Subject：Intracerebral hemorrhage）OR（Subject：Ischemic stroke）OR（Subject：Hemorrhagic stroke）AND（Subject：Acute Kidney Injury）OR（Subject：Acute Renal Injury）OR（Subject：Acute Renal Failures）OR（Subject：Acute Renal Insufficiencies）AND（Subject：Predict）OR（Subject：Models）OR（Subject：Risk Assessment）OR（Subject：Early warning）OR（Subject：Score）OR（Subject：Tools）

**8.CBM**

**Search Date:**April 26,2025

**Search Strategy:**

**#1** ( "中风" OR "卒中" OR "脑梗" OR "脑栓塞" OR "缺血性卒中" OR "出血性卒中" OR "脑出血" OR "脑血管意外")

**#2** "急性肾损伤" OR "急性肾损害" OR "急性肾功能衰竭" OR "急性肾功能不全"

**#3** "预测" OR "模型" OR "风险评估" OR "预警" OR "评分" OR "工具"

**#4** (#3) AND (#2) AND (#1)

**Results:**A total of 159 records were initially retrieved.

**Translation:**

**#1** ( "Apoplexy" OR "Stroke" OR "Cerebral infarction" OR "Brain infarction" OR "Ischemic stroke" OR "Hemorrhagic stroke" OR "Intracerebral hemorrhage" OR "Cerebrovascular accident")

**#2** "Acute Kidney Injury" OR "Acute Renal Injury" OR "Acute Renal Failures" OR "Acute Renal Insufficiencies"

**#3** "Predict" OR "Models" OR "Risk Assessment" OR "Early warning" OR "Score" OR "Tools"

**#4** (#3) AND (#2) AND (#1)

**Hand-search steps:**We manually checked the list of relevant literature included in the study to identify potential supplementary literature that might meet the requirements, and used Google Scholar for citation tracking to review papers that cited these key literature(e.g. doi: 10.1007/s11255-023-03646-6 DOI:10.2147/RMHP.S335150 doi:10.1161/STROKEAHA.125.051457). Through this method, we identified three pieces of literature.
