# Supplementary material for: Prediction Models for Acute Kidney Injury in Stroke Patients: A Systematic Review
Source: Brain Behav. 2026 Jan 7;16(1):e71188. doi: 10.1002/brb3.71188 (PMC12778413; doi:10.1002/brb3.71188)
Supplement: Supplementary file 2 — Supplementary Table: brb371188‐sup‐0002‐TableS2.docx [file BRB3-16-e71188-s001.docx]

**The item-level rationales：**

In the participant domain , fourteen studies were assessed as having a high risk of bias, primarily due to the use of inappropriate data sources (Arora et al.,2024;Kim et al.,2014;Zhang et al.,2022;Liu et al.,2022;Lu et al.,2024;Ma et al.,2024;Zhu et al.,2022;Tian et al.,2023;She et al.,2023;Liu et al.,2023;Xue et al.,2024;Xiao et al.,2024;An et al.,2023;He et al.,2024). In the predictor domain, one study was rated as having an unclear risk of bias because it did not report whether the predictors were blinded during the development or application of the prediction model (Arora et al.,2024). Five studies exhibited a high risk of bias in this domain, as outcome information was used in the assessment of predictors (Zhang et al.,2022;Tian et al.,2023;Xue et al.,2024;Zhang et al.,2023;He et al.,2024).

In the outcome domain, one study failed to report information on outcome classification (Arora et al.,2024), while eight studies incorporated predictor information in the definition of the outcome (Zhang et al.,2022;Lu et al.,2024;Ma et al.,2024;Zhu et al.,2022;Tian et al.,2023;She et al.,2023;An et al.,2023;He et al.,2024). In the statistical analysis domain, two studies were deemed to have an insufficient sample size, with events per variable (EPV) below 10 (Arora et al.,2024;Kim et al.,2014). Ten studies did not explicitly describe the handling of missing data (Arora et al.,2024;Kim et al.,2014;Liu et al.,2023;Zhu et al.,2022;She et al.,2023;Liu et al.,2023;Xue et al.,2024;Xiao et al.,2024;An et al.,2023;He et al.,2024),and two studies applied complete-case analysis without justification (Zhang et al.,2022;Tian et al.,2023). Five studies did not appropriately evaluate the predictive performance of the model (Arora et al.,2024;Kim et al.,2014;Liu et al.,2023;Ma et al.,2024;She et al.,2023). Five studies did not employ any internal validation method (Arora et al.,2024;Kim et al.,2014;Liu et al.,2023;Rao et al.,2022;An et al.,2023), and four relied solely on random split-sample validation (Ma et al.,2024;Zhu et al.,2022;Tian et al.,2023;He et al.,2024).

In terms of applicability risk assessment, 15 studies were rated as high risk in the participant domain due to the inclusion of only specific subgroups of stroke patients (Arora et al.,2024;Kim et al.,2014;Zhang et al.,2022;Liu et al.,2022;Lu et al.,2024;Ma et al.,2024;Zhu et al.,2022;Tian et al.,2023;She et al.,2023;Liu et al.,2023;Xue et al.,2024;Zhang et al.,2023;Xiao et al.,2024;An et al.,2023;He et al.,2024). In the domains of predictors and outcomes, two studies was rated as having high concern because acute kidney injury (AKI) was assessed using definitions that differed from the established criteria (Kim et al.,2014;Liu et al.,2023).
