# Supplementary material for: Prediction Models for Acute Kidney Injury in Stroke Patients: A Systematic Review
Source: Brain Behav. 2026 Jan 7;16(1):e71188. doi: 10.1002/brb3.71188 (PMC12778413; doi:10.1002/brb3.71188)
Supplement: Supplementary file 3 — Supplementary Table: brb371188‐sup‐0003‐TableS3.docx [file BRB3-16-e71188-s004.docx]

**Table 2.** Model predictors and performance

| Author(year) | Predictors | Missing data  handling | Model  presentation | Model validation | | Discrimination | | Calibration | DCA |
| --- | --- | --- | --- | --- | --- | --- | --- | --- | --- |
|  |  |  |  | Internal  validation | External  validation | AUC(95% CI)in  the development  cohorts | AUC(95% CI)in  the validation  cohorts |  |  |
| Arora et al.  2024 | Ischemic stroke: Age, sex, mechanical ventilation, tracheotomy, history of hypertension, admission-NIHSS, glomerular filtration rate  Hemorrhagic stroke: Age, sex, mechanical ventilation, tracheotomy, diabetes mellitus, history of hypertension, smoking, alcohol use, admission-NIHSS, glomerular filtration rate | — | — | — | — | — | — | — | — |
| Kim et al.  2014 | Age, hypertension, mannitol infusion rate, glomerular filtration rate | — | Rating scale | — | — | 0.917(0.851-0.983) | — | — | — |
| Zhang et al.  2022 | Serum creatinine levels, hemoglobin, WBC, bicarbonate, bloodurea nitro-gen, sodium, albumin, platelet count, age, length of hospital stay | Complete case  analysis | — | 5-fold cross-validation | Temporal validation | — | 0.880/-  0.780/-  0.870/-  0.850/-  0.780/-  0.780/-  0.790/-  0.780/-  0.780/-  0.670/- | Calibration curve | — |
| Liu et al.  2022 | WBC to lymphocyte ratio, WBC  to basophils ratio, WBC to hemoglobin ratio, neutrophil to lymphocyte ratio | — | Risk scoring formula | — | Geographical validation | 0.779 | — | — | — |
| Lu et al.  2024 | Drinking habits, respiratory rate, mechanical ventilation, serum creatinine levels, pulmonary infection, hemiplegia, diabetes mellitus, hypertension, NIHSS score, total cholesterol, low-density lipoprotein, urea nitrogen, blood potassium, glomerular filtration rate | multiple imputation | SHAP | 5-fold cross-validation | Temporal validation | 1.000  0.962(0.954-0.970)  1.000  1.000  0.971(0.964-0.977)  0.933(0.922-0.944)  0.903(0.889-0.917)  0.906(0.892-0.919)  0.938(0.928-0.948) | 0.955(0.937-0.972)  0.955(0.937-0.973)  0.949(0.930-0.969)  0.941(0.919-0.963)  0.953(0.935-0.972)  0.933(0.910-0.955)  0.895(0.866-0.924)  0.911(0.885-0.937)  0.859(0.825-0.894) | Calibration curve | Net benefit demonstrated between 10%-70% |
| Ma et al.  2024 | Weight, prior congestive heart failure, GCS, urine output, heart rate, blood glucose level, WBC, blood calcium concentration, vasoactive drugs injection, furosemide administration, invasive mechanical ventilation, supple-mental oxygen | multiple imputation | Nomogram model | Random split | — | — | — | Calibration curve and  Brier Score (0.157) | Net benefit demonstrated between 20%-100% |
| Zhu et al.  2022 | Blood urea nitrogen, creatinine, red blood cell distribution width, heart rate, Oxford Acute Severity of Illness Score, the history of congestive heart failure, the use of vancomycin, contrast agent, mannitol | — | Nomogram model | Random split | — | 0.8529(0.8036-0.8954) | 0.8598(0.8017-0.8806) | Hosmer-Lemeshow test P=0.8814 and calibration curve | Net benefit demonstrated in the range of 0-100% and 0-64% |
| Tian et al.  2023 | Gender, systolic blood pressure, diabetes mellitus, GCS, mannitol infusion, serum creatinine levels, albumin, uric acid, neutrophil/lymphocyte cell ratio | Complete case  analysis | Calculators | Random split | Geographical validation | 0.815 (0.796-0.833) | 0.816(0.788-0.843)  0.776(0.739-0.814)  0.780(0.745-0.815)  0.821(0.763-0.878) | Hosmer-Lemeshow test P=0.304  and calibration curve | — |
| She et al.  2023 | Platelet count, serum creatinine levels, vancomycin level, hemoglobin level, hematocrit level | — | SHAP | 5-fold cross-validation | — | 0.846(0.816-0.875)  0.698(0.659-0.737)  0.535(0.500-0.570)  1.000  0.810(0.778-0.841)  0.428(0.383 -0.472) | — | — | — |
| Liu et al.  2023 | Age ≥60 years, comorbid hypertension, Ultrasensitive C-reactive protein ≥15.8 mg/L | — | Nomogram  model | Bootstrap | — | 0.885(0.800-0.969) | — | Hosmer-Lemeshow test P=0.508 | — |
| Xue et al.  2024 | Combined acute respiratory failure, elevated blood urea nitrogen, d-dimer, monocyte count levels, antibiotics, diuretic use, mechanical ventilation, mannitol use | — | Nomogram model | Bootstrap | Geographical validation | 0.877(0.844-0.910) | 0.875(0.844-0.911) 0.798(0.679-0.917) | Calibration curve and  Brier Score(0.089) | Net benefit demonstrated between 10%-100% |
| Zhang et al.  2023 | Elevated neutrophils, prolonged prothrombin time, elevated lactate dehydrogenase, decreased glomerular filtration rate, history of blood transfusion, comorbid chronic kidney disease, use of antibiotics, use of disulfiram, diuretics use, use of beta-blockers | multiple imputation | Nomogram model | Bootstrap | — | 0.797(0.769-0.866) | 0.762(0.761-0.762) | Calibration curve | Net benefit demonstrated between 10%-60% |
| Xiao et al.  2024 | Preoperative GCS, ASA grade >3, heart rate at ICU admission | — | Nomogram model | Bootstrap | — | 0.795(0.727-0.863) | — | Hosmer-Lemeshow test P=0.376 | — |
| Rao et al.  2022 | Sex, history of hypertension, NIHSS score, history of collaterals diuretic use, history of mechanical thrombolysis, serum β2-microglobulin, urea nitrogen, serum cystatin C | Mean/Median Imputation | Regression equation | — | Temporal validation | 0.916(0.891-0.940) | 0.906(0.853-0.960) | Hosmer-Lemeshow test P=0.367 | — |
| An et al.  2023 | Younger group: Anemia, systolic blood pressure, homocysteine, alcohol consumption, blood urea nitrogen, NIHSS score  Middle-aged and older group: Hypertension, atrial fibrillation, previous history of stroke, cigarette smoking, infections, triglycerides, NIHSS score, use of antihypertensives, diuretics use, serum creatinine levels, blood urea nitrogen | — | — | — | Temporal validation | 0.938(0.912-0.963)  0.838(0.808-0.868) | — | Hosmer-Lemeshow test P=0.345 P=0.188 | — |
| He et al.  2024 | Age, weight, heart rate, blood creatinine, invasive ventilation, vascular catheterization, heart failure, albumin, vancomycin medication use, GCS | — | Nomogram model | Random split | — | 0.78 | 0.80 | Hosmer-Lemeshow test P =0.473  and calibration curve | Net benefit demonstrated between 30%-100% |

Note: "-" indicates not reported; ASA: the American Society of Anesthesiologists grade; NIHSS: the National Institutes of Health Stroke Scale; WBC: White Blood Cell; GCS: Glasgow Coma Scale; ICU: Intensive Care Unit; SHAP: SHapley Additive exPlanations; A: development cohort; B: validation cohort; Hosmer-Lemeshow test: Hosmer-Lemeshow goodness-of-fit test.
