# Supplementary material for: Prediction Models for Acute Kidney Injury in Stroke Patients: A Systematic Review
Source: Brain Behav. 2026 Jan 7;16(1):e71188. doi: 10.1002/brb3.71188 (PMC12778413; doi:10.1002/brb3.71188)
Supplement: Supplementary file 4 — Supplementary Table: brb371188‐sup‐0004‐TableS4.docx [file BRB3-16-e71188-s002.docx]

**The 12 most common predictors across the 16 studies.**

| **Predictors** | **Occurrences** |
| --- | --- |
| hypertension | 6 |
| serum creatinine levels | 6 |
| age | 6 |
| Diuretics use | 5 |
| mechanical ventilation | 5 |
| the National Institutes of Health Stroke Scale score | 4 |
| glomerular filtration rate | 4 |
| Glasgow Coma Score | 4 |
| alcohol use | 3 |
| heart failure | 3 |
| albumin | 3 |
| vancomycin medication use | 3 |
